# Proteasome Inhibitor Bortezomib Suppresses Nuclear Factor-Kappa B Activation and Ameliorates Eye Inflammation in Experimental Autoimmune Uveitis

**DOI:** 10.1155/2015/847373

**Published:** 2015-01-12

**Authors:** Sheng-Min Hsu, Chang-Hao Yang, Fang-Hsiu Shen, Shun-Hua Chen, Chia-Jhen Lin, Chi-Chang Shieh

**Affiliations:** ^1^Institute of Clinical Medicine, College of Medicine, National Cheng-Kung University, No. 35, Siao-Dong Road, Tainan 70403, Taiwan; ^2^Department of Ophthalmology, National Cheng-Kung University Hospital, Tainan 70403, Taiwan; ^3^Department of Ophthalmology, National Taiwan University Hospital, College of Medicine, National Taiwan University, Taipei 10051, Taiwan; ^4^Department of Microbiology and Immunology, College of Medicine, National Cheng-Kung University, Tainan 70101, Taiwan; ^5^Department of Pediatrics, National Cheng-Kung University Hospital, Tainan 70403, Taiwan

## Abstract

Bortezomib is a proteasome inhibitor used for hematologic cancer treatment. Since it can suppress NF-*κ*B activation, which is critical for the inflammatory process, bortezomib has been found to possess anti-inflammatory activity. In this study, we evaluated the effect of bortezomib on experimental autoimmune uveitis (EAU) in mice and investigated the potential mechanisms related to NF-*κ*B inactivation. High-dose bortezomib (0.75 mg/kg), low-dose bortezomib (0.15 mg/kg), or phosphate buffered saline was given after EAU induction. We found that the EAU is ameliorated by high-dose bortezomib treatment when compared with low-dose bortezomib or PBS treatment. The DNA-binding activity of NF-*κ*B was suppressed and expression of several key inflammatory mediators including TNF-*α*, IL-1*α*, IL-1*β*, IL-12, IL-17, and MCP-1 was lowered in the high-dose bortezomib-treated group. These results suggest that proteasome inhibition is a promising treatment strategy for autoimmune uveitis.

## 1. Introduction

Uveitis is among the most important causes of blindness and severe visual impairment worldwide. About 15 to 30% of uveitis occurs in the choroid and adjacent retina and hence is classified as posterior uveitis or uveoretinitis [1]. Posterior uveitis tends to damage the photoreceptor cells and lead to permanent blindness. This severe intraocular inflammatory disease is often associated with autoimmune responses to unique retinal proteins [2]. Current therapies for uveitis are based largely on immunosuppressive treatment including corticosteroids, antimetabolites, and alkylating agents. Due to the nonspecific nature and the dose-limiting side effects of these drugs, the results of current treatment for autoimmune-mediated uveitis remain unsatisfactory [3]. Novel approaches to control the inflammatory process in uveitis hence are being keenly developed both in humans and in animal models [4].

Experimental autoimmune uveitis (EAU), in which eye inflammation is induced by active immunization with retinal antigens, is the most often used rodent model for the study of autoimmune uveitis [5]. The typical histological appearance of EAU resembles that of human posterior uveitis, with inflammatory cells infiltrating the vitreous cavity, retina, and choroid and causing damage to the photoreceptor cell layer [3]. Nuclear factor-kappa B (NF-*κ*B) has a pivotal role in inducing inflammation. Several previous studies have shown that there is an increased NF-*κ*B activation in EAU, and the inhibition of NF-*κ*B can ameliorate inflammation [6, 7]. Several NF-*κ*B-regulated inflammatory mediators, including interleukin- (IL-) 1, IL-6, tumor necrosis factor- (TNF-) *α*, interferon- (IFN-) *γ*, monocyte chemoattractant protein- (MCP-) 1, and inducible nitric oxide synthase (iNOS), were found to increase in animals with EAU and may be modulated by treatment targeting NF-*κ*B [3].

The degradation of ubiquitinated I*κ*B by the proteasome is important for the activation of NF-*κ*B [8, 9]. Meanwhile, inhibition of NF-*κ*B activation has been shown to be beneficial in animal models of experimental autoimmune disease, such as myasthenia gravis, psoriasis, arthritis, and autoimmune encephalomyelitis [10–13]. However, little is known about the effectiveness of proteasome inhibition in treating autoimmune uveitis. Here, we showed the effectiveness of bortezomib, a 26S proteasome inhibitor, in inhibiting IRBP-induced EAU.

## 2. Materials and Methods

### 2.1. Mice

Female C57BL/6J (B6) mice (8- to 12-weeks-old) were obtained from the Laboratory Animal Center at the National Cheng-Kung University and used for all experiments. All experiments were performed in compliance with a protocol approved by the Institutional Animal Care and Use Committee of the National Cheng-Kung University and with the ARVO Statement for the Use of Animals in Ophthalmic and Vision Research.

### 2.2. Induction and Treatment of EAU

EAU was induced as previously described with modifications [14]. Briefly, mice were immunized with 100 *μ*L of an emulsion of phosphate buffered saline (PBS) containing 200 *μ*g of human IRBP peptide 1–20 (hIRBP_1–20_) (GPTHLFQPSLVLDMAKVLLD) and complete Freund's adjuvant (CFA) containing 500 *μ*g of inactivated* Mycobacterium tuberculosis* H37RA (Difco Laboratories, Detroit, MI, USA). Mice received the emulsion at two sites on the lower back, followed by an intraperitoneal (i.p.) injection of 1.5 *μ*g pertussis toxin (PTX) as an additional adjuvant. Mice were treated with PBS, bortezomib (Millennium Pharmaceuticals, Cambridge, MA) at the doses of 0.75 or 0.15 mg/kg (Velcade (H) and (L) groups, resp.), or etanercept (Enbrel, Wyeth Pharmaceuticals, Hampshire, UK) at the dose of 5 mg/kg in 0.1 mL by i.p. injection twice a week starting on the day of EAU induction.

### 2.3. Clinical Scoring of EAU

Ocular fundus of the mouse eyes was examined by slit lamp twice a week from the 7th day after induction until the end of experiments for clinical signs of EAU. Pupils were dilated using tropicamide and phenylephrine hydrochloride ophthalmic solutions. The severity of inflammation was clinically graded on a scale of 1–5 as described previously [15]. Briefly, 0 = no inflammation; 1 = focal vasculitis ≤ 5 spots or soft exudates ≤ 5; 2 = linear vasculitis or spotted exudates < 50% of the retina; 3 = linear vasculitis or spotted exudates ≥ 50% of the retina; 4 = retinal hemorrhage or severe exudates and vasculitis; 5 = exudative retinal detachment or subretinal (or vitreous) hemorrhage. A mouse was considered to have uveitis if at least one of its eyes had a score of two or more. The severity of uveitis is represented as the highest clinical score achieved by either eye in a mouse.

### 2.4. Histopathological Evaluation

Whole eyes were collected at the peak of the clinical response (21 days after induction of EAU), immersed in 10% formaldehyde, and then stored until being processed. Fixed and dehydrated tissues were embedded in paraffin and 3 *μ*m sections were cut through the cornea-optic nerve plane and then stained with hematoxylin and eosin (H&E). Presence or absence of disease was evaluated in a blinded fashion by examining six sections cut at different levels for each eye. The severity of inflammation was histologically graded on a scale of 1–4 as described previously [16]. Briefly, 0 means no change; 1 means mild cell infiltration and focal retinal folds; 2 means moderate cell infiltration and retinal folds; 3 means moderate to heavy cell infiltration and extensive retinal folding with detachments; 4 means heavy cell infiltration with diffuse retinal detachment. Therefore, leukocytes infiltration into the vitreous cavity and retinal folding were considered as posterior uveitis.

### 2.5. Preparation of Retinal Lysate for Luminex Analysis

The eyes were enucleated from euthanized mice. The eyeballs were cut at the equator around the ora serrata, and the posterior pole of the eyes was separated from the anterior pole and lens. From the posterior pole, the neurosensory retina was extracted from retinal pigment epithelial layer. The extract from six retinas was placed in 300 *μ*L of 0.5% NP-40 (Abcam) on ice (one minute) and briefly sonicated five times for 10 seconds at probe intensity of 7 (MicrosonTM XL2000 Ultrasonic liquid processor, Qsonica, LLC, Newton, CT). After removal of the insoluble material by centrifugation (200 ×g for 5 min), the protein concentration of the retinal extract was measured at 280 nm on ND-1000 Spectrophotometer. Then, the retinal lysate was used for Luminex analysis as below.

### 2.6. Analysis of Inflammatory Mediators in Retinas by Luminex

Quantification of TNF-*α*, IFN-*γ*, IL-1*α*, IL-1*β*, IL-4, IL-6, IL-12, IL-17, and MCP-1 in retinal tissues was carried out using murine multiplexing bead immunoassays (Invitrogen, Carlsbad, CA) according to manufacturer's instruction. Briefly, 25 *μ*L of retinal samples in PBS was incubated with antibody-coupled beads. After series of washes, a biotinylated detection antibody was added to the beads, and the reaction mixture was detected by the addition of streptavidin-phycoerythrin. The bead set was analyzed using a flow-based Luminex 200 suspension array system (Luminex Corporation, Austin, TX, USA).

### 2.7. Measurement of Proteasome Activity in the Retina

The chymotrypsin-like and trypsin-like activity of the proteasome of the retinas in the bortezomib or PBS-treated mice which were sacrificed 21 days after EAU induction was determined using commercial proteasome assay kits (Proteasome-Glo assay systems; Promega) according to the manufacturer's instructions. Briefly, the Suc-LLVY-Glo substrate (for chymotrypsin-like activity) or Z-LRR-Glo substrate (for trypsin-like activity) was added to the mixture of the Proteasome-Glo buffer and the luciferin detection reagent and incubated at room temperature for 1 hour. The retinal tissue was minced in 100 *μ*L of ice-cold PBS containing 5 mM EDTA followed by centrifugation at 12,000 g at 4°C for 10 minutes. A 50 *μ*L of retinal sample was added by equal volume of reagent mixture and incubated for 90 minutes. Finally the luminescence of retinal sample was detected by a microplate luminometer (Promega).

### 2.8. Nuclear Protein Extract and Electrophoretic Mobility Shift Assay (EMSA) of NF-*κ*B

Nuclear protein extracts were obtained as described previously [17]. Briefly, the retinas were minced in 0.5 mL of lysis buffer (10 mM HEPES, 1.5 mM KCl, 10 mM MgCl_2_, 1.0 mM DTT, and 1.0 mM PMSF). The tissue was homogenized, followed by centrifugation at 5,000 g at 4°C for 10 minutes. The sediment was suspended in 200 *μ*L of extraction buffer (20 mM HEPES, 25% glycerol, 1.5 mM MgCl_2_, 420 mM NaCl, 0.5 mM DTT, 0.2 mM EDTA, 0.5 mM PMSF, and 4 *μ*M leupeptin), and the suspension was incubated on ice for 30 minutes. The sample was then centrifuged at 12,000 g at 4°C for 30 minutes. The supernatant containing the nuclear proteins was collected and stored at −70°C until use. The protein concentration was determined with a bicinchoninic acid assay kit (Pierce Biotechnology, Rockford, IL). The EMSA was performed with an NF-*κ*B DNA-binding protein detection system (Pierce Biotechnology) according to the manufacturer's instructions. A 10 *μ*g nuclear protein was incubated with a biotin-labeled NF-*κ*B consensus oligonucleotide probe (5′-AGTTGAGGGGACTTTCCCAGGC-3′) for 30 minutes in binding buffer. The specificity of the DNA protein binding was determined by adding a 100-fold molar excess of unlabeled NF-*κ*B oligonucleotide for competitive binding 10 minutes before adding the biotin-labeled probe.

### 2.9. Statistical Analysis

Values are shown as the mean ± SD. For statistical comparison, data were analyzed by the Wilcoxon signed-rank test, Student's *t*-test, or Chi-square test using Prism 5.0 software. In all tests, *P* values less than 0.05 were considered statistically significant.

## 3. Results

### 3.1. A High Dose of Bortezomib Significantly Decreased Uveoretinitis in EAU Mice

**Figure 1:**
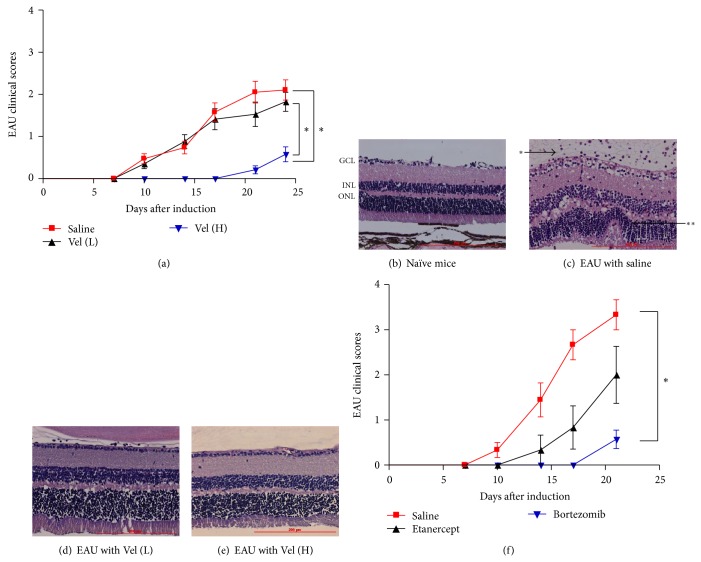
Effect of bortezomib on clinical course of EAU induced with IRBP. (a) Comparison of clinical scores of EAU mice treated with high-dose (0.75 mg/kg) bortezomib (red line, *n* = 19), low-dose (0.15 mg/kg) bortezomib (black line, *n* = 17), or PBS (blue line, *n* = 19) in 0.1 mL. Data shown are the mean clinical score (ordinate) of each experiment group over time (abscissa) and the sum of three independent experiments. Comparison of (the course of the clinical symptoms) high-dose bortezomib-treated EAU mice (blue line) with saline-treated mice (red line) shows a significant difference and is indicated as (^*^). Comparison of high-dose bortezomib-treated (blue line) with low-dose bortezomib-treated (black line) EAU mice also shows a significant difference and is indicated as (^*^). ^*^ *P* < 0.05, via the Wilcoxon signed-rank test. (b), (c), (d), and (e): photomicrographs of H&E stained retinal tissue. Representative photomicrographs of paraffin-fixed H&E stained slides of the retina of (b): naïve C57BL/6 mice without EAU induction, (c): EAU mice that received 0.1 mL PBS treatment (^*^ indicates leukocytes in vitreous cavity; ^**^ indicates retinal folds), (d): EAU mice that received low-dose (0.15 mg/kg) bortezomib treatment, and (e): EAU mice that received high-dose (0.75 mg/kg) bortezomib treatment. The experiment was repeated three times with similar results. GCL: ganglion cell layer. INL: inner nuclear layer. ONL: outer nuclear layer. (f) Average clinical score over time of EAU in mice with high-dose (0.75 mg/kg) bortezomib (blue line, *n* = 9), etanercept (5 mg/kg) (black line, *n* = 6), or saline (0.1 mL/mouse) treatment (red line, *n* = 7). Data shown are the mean clinical score (ordinate) of each experiment group over time (abscissa) and the sum of two independent experiments. ^*^ *P* < 0.05, via the Wilcoxon signed-rank test.

EAU was induced in mice by injecting 200 *μ*g of IRBP_1–20_ emulsified with CFA subcutaneously and 1.5 *μ*g of pertussis toxin (PTX) intraperitoneally as described in Section 2. At the same time, high- (0.75 mg/kg) and low-dose (0.15 mg/kg) bortezomib were injected intraperitoneally into mice and then twice a week until the end of the experiments. A group of mice which received PBS instead of bortezomib served as controls. In PBS-treated group, the disease showed sign of inflammation 9–15 days later and developed over the following 4-5 days when it reached the peak. Mice that received IBRP_1–20_ immunization plus treatment with high-dose bortezomib exhibited a significant delay in disease onset and a significantly lower peak EAU score over time (Figure 1(a), Table 1). While both the saline-treated and low-dose bortezomib-treated mice had higher incidence of disease (14 of 19 and 10 of 17, resp.), we observed that the high-dose bortezomib-treated mice had a significantly lower incidence of EAU (3 of 19, *P* < 0.05) (Table 1). The mice that received saline treatment had a mean clinical severity score of 2.16 ± 0.25 while the mice that received high-dose bortezomib treatment had a mean clinical severity score of 0.58 ± 0.18 (*P* < 0.05) (Table 1). The mice that received low-dose bortezomib treatment showed a slightly lower mean clinical severity score of 2.00 ± 0.23, which is not significantly different from that of the saline-treated control group. The fact that the majority of mice given high-dose bortezomib had peak scores of 1 or lower (i.e., mild or no disease) indicated a suppressive activity of bortezomib on EAU. In addition, examination of H&E stained paraffin fixed slides revealed that retinal sections of eyes from EAU mice that received high-dose bortezomib had a reduced cell infiltration into the vitreous cavity and their retinal layer structures lacked the retinal folds observed in the saline-treated mice (Figures 1(b)–1(e)). There was no mortality or extraocular morbidity associated with the bortezomib treatment in the experimental animals. The body weight and the level of hemoglobin of the mice did not differ significantly between the saline- and bortezomib-treated groups at the end of experiment (data not shown). There was also no tumor growth or infection after bortezomib treatment in our study.

### 3.2. Bortezomib Treatment Suppressed EAU More than TNF-Alpha Antagonist Treatment

Previous studies showed that TNF-*α* antagonist could also suppress uveitis in human and mice [18, 19]. We hence compared the effect of suppression of EAU by bortezomib or TNF-*α* antagonist etanercept. Bortezomib (0.75 mg/kg) or etanercept (5 mg/kg) was injected into EAU mice twice a week from the day of EAU induction. A group of mice that received PBS (0.1 mL/mouse) served as controls. Mice that received IRBP_1–20_ immunization plus treatment with bortezomib exhibited a significant delay in disease onset and a significantly lower peak EAU score over time (Figure 1(f), Table 2). The mice that received treatment with etanercept also had lower incidence and mean peak disease score. However, the differences between saline- and etanercept-treated groups did not reach statistical significance (Table 2, *P* = 0.06). Therefore, treatment with bortezomib suppressed the development and severity of EAU more effectively than the TNF-*α* antagonist etanercept. There was no mortality, morbidity, tumor growth, or infection associated with the bortezomib or etanercept treatment in the EAU mice at the end of experiment.

### 3.3. The Influence of Bortezomib on the Levels of Inflammatory Mediators in Retina of EAU Mice

**Figure 2:**
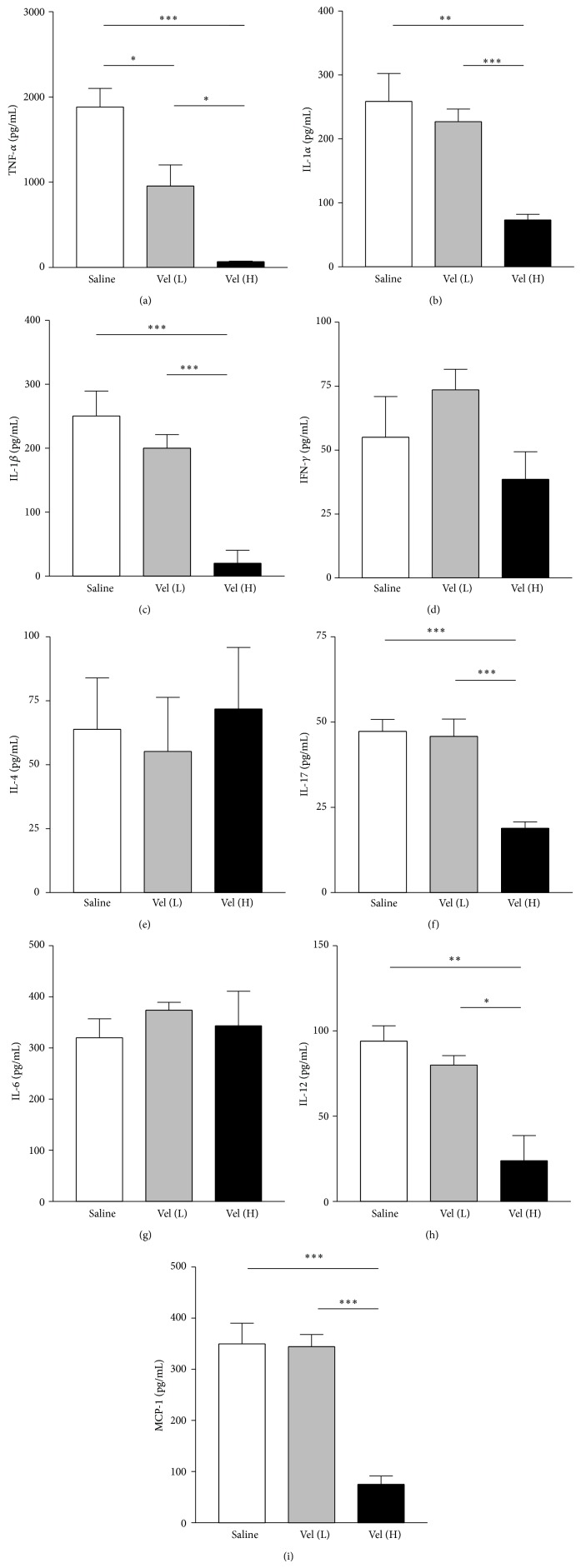
The evaluation of protein expression of inflammatory mediators in retinas of EAU mice in Luminex analysis. Decreased expression of TNF-*α* (a), IL-1*α* (b), IL-1*β* (c), IL-17 (f), IL-12 (h), and MCP-1 (i) relative to the expression in the saline-treated group was noted in the high-dose bortezomib (Vel [H]) group but not in the low-dose bortezomib (Vel [L]) group except for TNF-*α*. In addition, there was no significant difference on the expression of IFN-*γ* (d), IL-4 (e), and IL-6 (g) in retinas between bortezomib and saline-treated mice. Data are expressed as the mean SD of three independent experiments (bar graph). ^*^ *P* < 0.05, ^**^ *P* < 0.01, and ^***^ *P* < 0.001, via Student's *t*-test.

We then measured the cytokine levels in retinas in EAU mice with different treatment. When comparing the saline-treated group with low-dose bortezomib group, we found that TNF-*α* level was lower in the low-dose bortezomib-treated group, while the levels of other cytokines were not significantly different (Figures 2(a)–2(i)). Meanwhile, the levels of TNF-*α*, IL-1*α*, IL-1*β*, IL-12, IL-17, and MCP-1 in retina were significantly lower in the EAU mice treated with high-dose bortezomib when compared with saline-treated mice (*P* < 0.05 in all paired comparisons) (Figures 2(a), 2(b), 2(c), 2(f), 2(h), and 2(i)). However, there was no significant difference noted in IFN-*γ*, IL-4, and IL-6 between high-dose bortezomib-treated and saline-treated EAU mice (Figures 2(d), 2(e), and 2(g)). When comparing the groups treated with high-dose and low-dose bortezomib, we found that the levels of TNF-*α*, IL-1*α*, IL-1*β*, IL-12, IL-17, and MCP-1 were lower in the high-dose bortezomib group in comparison with the low-dose bortezomib group (*P* < 0.05 in all paired comparisons) (Figures 2(a), 2(b), 2(c), 2(f), 2(h), and 2(i)).

### 3.4. Bortezomib Treatment Significantly Reduced the Proteasome Activity of EAU Mice

**Figure 3:**
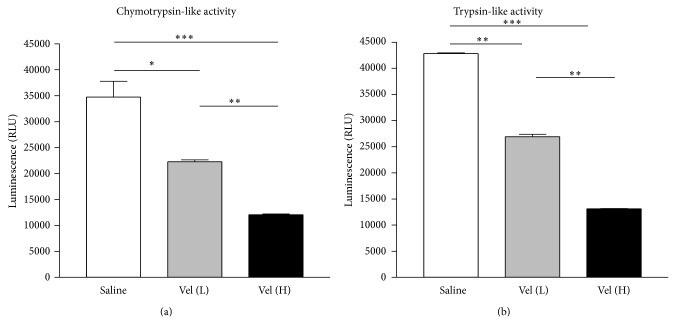
Evaluation of chymotrypsin-like and trypsin-like activity of the proteasome. Compared to the saline-treated EAU group, there was significantly decreased activity of chymotrypsin-like (a) and trypsin-like activity (b) in the low-dose [Vel (L)] and high-dose bortezomib [Vel (H)] groups. The activity was also markedly lowered in the high-dose bortezomib-treated group compared with the low-dose bortezomib-treated group. The data are expressed as the mean ± SD of the mean in 5 mice for each group (bar graph). ^*^ *P* < 0.05, ^**^ *P* < 0.01, and ^***^ *P* < 0.001, via Student's *t*-test. The experiment was repeated three times with similar results.

We then performed proteasome protease activity assays to evaluate the suppressive effect of bortezomib treatment in retinal tissue. The signal of luminescence indicates chymotrypsin-like or trypsin-like activity in the retinal tissues of EAU mice. The signals were significantly lower in the high-dose bortezomib-treated group when compared with the saline or low-dose bortezomib-treated group (*P* < 0.05) (Figures 3(a) and 3(b)). There was also a significant difference in the signal of luminescence between the low-dose bortezomib and saline-treated groups (Figures 3(a) and 3(b)).

### 3.5. The Increased Binding of NF-*κ*B and DNA in EAU Mice Was Inhibited by Bortezomib Treatment

**Figure 4:**
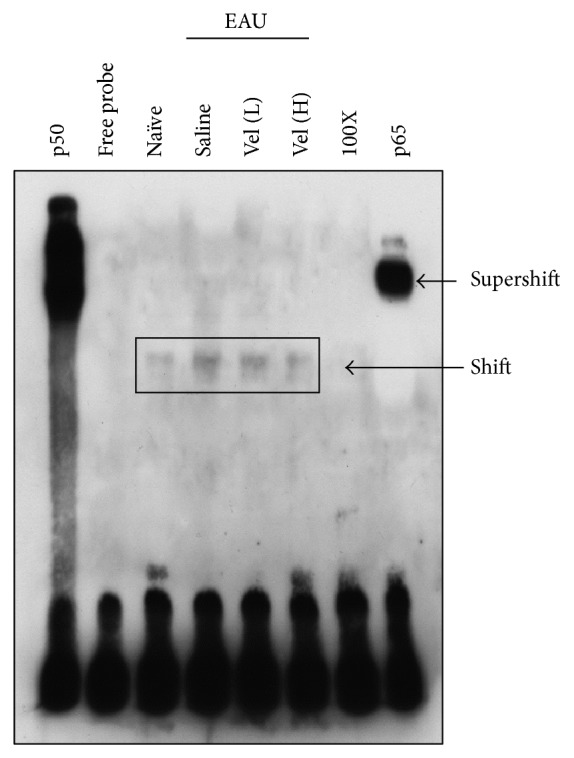
EMSA for the evaluation of the NF-*κ*B DNA-binding activity in naïve mice and different groups of EAU mice.* Lane 1*: p50 subunit of NF-*κ*B.* Lane 2*: free probe (FP).* Lane 3*: naïve C57BL/6 mice.* Lane 4*: EAU mice with saline treatment.* Lane 5*: EAU mice treated with low-dose bortezomib (Vel [L]).* Lane 6*: EAU mice treated with high-dose bortezomib (Vel [H]).* Lane 7*: 100-fold molar excess of unlabeled NF-*κ*B probe.* Lane 8*: anti-p65 subunit of NF-*κ*B. The sample was pooled from both eyes of five mice in each group. Data are representative of results in three independent experiments.

The involvement of NF-*κ*B pathway during bortezomib treatment in EAU was analyzed with EMSA. Compared to the naïve group, the NF-*κ*B DNA binding increased after EAU induction significantly (Figure 4, Shift of Naïve and Saline). The increased activity of NF-*κ*B DNA binding after EAU induction was markedly inhibited by treatment with low-dose bortezomib. High-dose bortezomib treatment further suppressed the NF-*κ*B DNA binding (Figure 4, Shift of Vel (L) and Vel (H)). Adding a 100-fold molar excess of unlabeled NF-*κ*B probe completely blocked the binding of the labeled probe to the NF-*κ*B DNA complex (Figure 4, Shift of 100X). Therefore, bortezomib reduced the binding of NF-*κ*B DNA in a dose-responsive manner. The results from protease inhibition (Figure 3) and NF-*κ*B DNA binding implicated that the activation of NF-*κ*B was effectively suppressed by proteasome inhibition.

## 4. Discussion

Our study demonstrated that bortezomib, a 26S proteasome inhibitor, is active in suppressing NF-*κ*B activation and is effective in inhibiting ocular inflammation and reducing the production of inflammatory mediators in EAU. Our results indicate that inhibition of proteasome may be a promising approach to treating autoimmune uveitis.

Recent evidence indicated that NF-*κ*B has a pivotal role in EAU and that the inhibition of NF-*κ*B activation can reduce the levels of tissue inflammation by lowering the inflammatory mediators and cell infiltration into the uvea [6, 7]. Since proteasomal degradation of the inhibitory factor I*κ*B is important for NF-*κ*B activation, the inhibition of proteasome maintains NF-*κ*B in the inactive state in the cytosol and prevents its nuclear translocation. Proteasome inhibition has been found to be effective in treating several animal models of autoimmune disease such as myasthenia gravis, psoriasis, arthritis, and autoimmune encephalomyelitis [10–13]. Moreover, Chen et al. have shown the anti-inflammatory effect of proteasome inhibitor on endotoxin-induced uveitis in rats [17]. The proteasome inhibitors bortezomib, due to their activity to suppress nonlysosomal protein degradation, has been used in the treatment of hematologic cancers in clinical settings [20]. In this study, we chose bortezomib based on its high efficacy at minimal concentrations and tolerable and manageable adverse effects in treating human hematologic diseases [21]. To our knowledge, our study is the first to demonstrate its anti-inflammatory effect in EAU.

Being an autoantigen-induced autoimmune condition, EAU has an inflammation dominated by acute inflammatory cytokine response [3]. In our study, we found that levels of inflammatory mediators including TNF-*α*, IL-1*α*, IL-1*β*, IL-12, IL-17, and MCP-1 increased significantly in saline-treated EAU mice when compared with those in naïve mice (Figure 2). TNF-*α* is a major proinflammatory cytokine and plays a central role in autoimmune uveitis [3]. TNF-*α* antagonists have been used clinically to treat ocular inflammatory disorders successfully [19, 22]. Therefore, in this study we compared the anti-inflammatory effect between proteasome inhibitor bortezomib and the TNF-*α* antagonist, etanercept, in EAU. Etanercept, a chimeric protein of human TNF-*α* receptor and Fc portion of immunoglobulin G heavy chain, can neutralize TNF-*α* and inhibit its proinflammatory activity in both humans and mice [18, 23, 24]. We found that bortezomib treatment in EAU mice could suppress not only TNF-*α* but also many other inflammatory mediators such as IL-1*α*, IL-1*β*, IL-12, IL-17, and MCP-1 in retinas so the autoimmune uveitis could be more effectively suppressed with bortezomib than with TNF-*α* antagonist etanercept (Figure 1(f)). Bortezomib, which may suppress multiple inflammatory cytokines through inhibiting NF-*κ*B activation, hence appears to be a better anti-inflammatory agent in treatment of autoimmune uveitis than TNF-*α* antagonists.

The mechanisms of the anti-inflammatory effects of bortezomib, however, may be more complex than the inhibition of NF-*κ*B activation. As the induction of EAU apparently involves the antigen presentation of the immunogenic antigen by major histocompatibility complex class I and class II molecules, the inhibition of proteasome, which is a critical component of the antigen processing, may also affect the autoimmune recognition process in sensitization and stimulation phases [25–29]. In addition, proteasome inhibitors have been demonstrated to trigger the apoptosis of leukocytic cells, which may contribute to their immunosuppressive and antitumor effect [30–33]. Proteasome inhibition hence may suppress several key steps necessary for activating the autoimmune responses in EAU.

In our study, the mice were treated with bortezomib from the same day when EAU was induced to ensure the onset of the drug's effect during early stage of the disease development. We have not evaluated the drug's efficacy when it is applied after EAU is full-blown, as usually is the case in clinic settings. At the end of experiment after bortezomib or etanercept treatment, there was no mortality, tumor growth, or severe infection noted. However, since the proteasome and its ubiquitous distribution regulate the wide range of biological functions, the systemic adverse effects associated with proteasome inhibitors deserve meticulous consideration.

In summary, we demonstrated that bortezomib ameliorated experimental autoimmune uveitis in mice in a dose-dependent manner. Reduced intraocular inflammation was associated with the inhibition of NF-*κ*B activation and decreased expression of many inflammatory mediators. Our encouraging results indicate that drugs targeting the proteasome may be an effective treatment strategy for autoimmune uveitis in the future.

## Figures and Tables

**Table 1:** Effect of high-dose versus low-dose bortezomib (Velcade) on EAU^a^.

Treatment	Incidence	Mean peak disease score
Saline	14/19	2.16 ± 0.25
Vel (L)^b^	10/17	2.00 ± 0.23
Vel (H)^c^	3/19^*^	0.58 ± 0.18^#,+^

^a^Data are compiled from three experiments in which similar results were obtained.

^b^Low-dose bortezomib 0.15 mg/kg ip treatment.

^c^High-dose bortezomib 0.75 mg/kg ip treatment.

^*^ *P* < 0.05, via the Chi-square test, between saline and Vel (H) groups.

^#^ *P* < 0.05, via the Wilcoxon signed-rank test, between saline and Vel (H) groups.

^+^ *P* < 0.05, via the Wilcoxon signed-rank test, between Vel (L) and Vel (H) groups.

**Table 2:** Effect of etanercept versus bortezomib (Velcade) on EAU.

Treatment	Incidence	Mean peak disease score
Saline	8/9	3.33 ± 0.33
Etanercept^a^	3/6	2.00 ± 0.63
Velcade^b^	0/7^*^	0.57 ± 0.20^#^

^a^Etanercept 5 mg/kg ip treatment.

^b^Bortezomib (Velcade) 0.75 mg/kg ip treatment.

^*^ *P* < 0.05, via the Chi-square test, between saline and bortezomib group.

^#^ *P* < 0.05, via the Wilcoxon signed-rank test, between saline and bortezomib group.

## Abbreviations

EAU: Experimental autoimmune uveitis
IRBP: Interphotoreceptor retinoid-binding protein
NF-*κ*B: Nuclear factor-kappa B.
